# The urgency of utilizing COVID-19 biospecimens for research in the heart of the global pandemic

**DOI:** 10.1186/s12967-020-02388-8

**Published:** 2020-06-01

**Authors:** Iman Osman, Paolo Cotzia, Una Moran, Douglas Donnelly, Carolina Arguelles-Grande, Sandra Mendoza, Andre Moreira

**Affiliations:** The New York University Langone Health (NYULH) Center of Biospecimen Research and Development, Office of Science and Research, NYU Grossman School of Medicine, 522 First Avenue, SML405, New York, NY 10016 USA

**Keywords:** Coronavirus disease 2019, Biobanking, Translational medicine

## Abstract

The outbreak of the novel coronavirus disease 2019 (COVID-19) and consequent social distancing practices have disrupted essential clinical research functions worldwide. Ironically, this coincides with an immediate need for research to comprehend the biology of severe acute respiratory syndrome coronavirus 2 (SARS-CoV-2) and the pathology of COVID-19. As the global crisis has already led to over 15,000 deaths out of 175,000 confirmed cases in New York City and Nassau County, NY alone, it is increasingly urgent to collect patient biospecimens linked to active clinical follow up. However, building a COVID-19 biorepository amidst the active pandemic is a complex and delicate task. To help facilitate rapid, robust, and regulated research on this novel virus, we report on the successful model implemented by New York University Langone Health (NYULH) within days of outbreak in the most challenging hot spot of infection globally. Using an amended institutional biobanking protocol, these efforts led to accrual of 11,120 patients presenting for SARS-CoV-2 testing, 4267 (38.4%) of whom tested positive for COVID-19. The recently reported genomic characterization of SARS-CoV-2 in the New York City Region, which is a crucial development in tracing sources of infection and asymptomatic spread of the novel virus, is the first outcome of this effort. While this growing resource actively supports studies of the New York outbreak in real time, a worldwide effort is necessary to build a collective arsenal of research tools to deal with the global crisis now, and to exploit the virus’s biology for translational innovation that outlasts humanity’s current dilemma.

## Introduction

The recent outbreak of the novel coronavirus disease 2019 (COVID-19) and the associated need for vital social practices that reduce further spread have disrupted clinical research functions worldwide [1]. Ironically, this interruption coincides with an especially critical need for human biospecimen research to better understand the biology of severe acute respiratory syndrome coronavirus 2 (SARS-CoV-2) and the pathology of COVID-19. As the global crisis has already led to over 15,000 deaths out of approximately 175,000 confirmed cases in New York City and Nassau County, NY alone [2], it is increasingly urgent to amass patient samples linked to prospective and active follow up.

Building a COVID-19 biorepository is a delicate and complex task that requires maximum biosafety measures and minimum interruption in an overburdened clinical delivery system. To help facilitate rapid, robust, and regulated research on this novel virus, we report on how the model implemented by New York University Langone Health (NYULH) led to prospective accrual of clinically linked research biospecimens from 11,120 patients presenting for SARS-CoV-2 within weeks. We also feature the earliest outcome of this pipeline, which is the recently reported genomic characterization linking the US and European viral strains, a crucial development in our efforts to combat COVID-19 [3].

## Methods

### Universal consent protocol

The NYU IRB-approved Universal consent (UC) protocol provides researchers with the infrastructure to collect human biospecimens and corresponding clinical data for research purposes at any NYULH facility at NYULH on an institution-wide level. The NYULH Center for Biospecimen Research and Development (CBRD) maintains ownership over all samples collected under the UC, until IRB-approved distribution.

Given the urgency of COVID-19 biospecimen collection and the consent-limiting clinical disease course, the IRB approved a temporary waiver of consent for enrollment in the UC study. For living patients, the waivered consent is effective until their clinical condition has stabilized and there is no added exposure risk on the patient and/or the research support staff by approaching for consent at the patients next clinical visit at NYULH or by adapting the current process to capture the patients consent or denial to use these specimens. If a patient denies consent, banked specimens will be destroyed, and any recorded data will be removed from the clinical database. Additionally, the waiver of consent permits the CBRD to bank de-identified leftover specimens and clinical data for patients who died before they can be approached to document the consent process.

The CBRD is an institutional biobank created in 2015 with the overarching goal to facilitate high-quality research on human biospecimens with linked clinicopathological information. The CBRD adopts the standards of and has accreditations from New York State Department of Health, the International Society for Biological and Environmental Repositories and the College of American Pathologists. The CBRD adheres to all biosafety level-3 guidelines for COVID-19 collections as outlined by the Center for Disease Control [4].

### COVID-19 collections and clinical database

The UC form (Additional file 1: Appendix S1) is automatically linked to the patient’s electronic medical record when completed and electronically signed. Biospecimens collected under the UC study are tracked using the Laboratory Information System known as Labvantage. Labvantage generates biospecimen labels with unique subject identification numbers for patients that sign the UC, manages parent and child biospecimen collection, and tracks clinical follow-up to notify CBRD staff of potential future collections.

To maximize COVID-19 collections, we modified this protocol to prospectively enroll all patients presenting to NYULH with a COVID-19 nasopharyngeal diagnostic test performed into Labvantage. Biospecimens were collected for all symptomatic and asymptomatic patients tested for the novel coronavirus. The clinical information from enrolled patients is extracted from the electronic medical record and recorded in 107 discrete demographic, medical, and COVID-19-specific data fields (Additional file 1: Appendix S1) in a REDCap [5] database.

## Discussion

Rapid accrual of biospecimens from research subjects tested for COVID-19 is essential to support the urgent need to both precisely characterize the biology of SARS-CoV-2 and to develop models that predict clinical outcomes accurately and reliably. The most efficient method to approve such a process at NYULH was to amend an existing protocol: the UC. The vital benefits of the UC for an institutional research program include enhancing patient protections and amassing population-level data, as non-clinical staff obtain consent and participation in the UC is institution-wide and not disease-specific. Using similar mechanisms will foster robust COVID-19 research by increasing the number of patients with different socioeconomic statuses, ethnic backgrounds, and medical histories, which will subsequently provide more generalizable results. By modifying the UC protocol to adapt to the current crisis, we built on the existing infrastructure developed at NYULH to maximize patient accrual, eliminate human error, and enable real-time data tracking in order to bank essential biospecimens required to further our understanding of COVID-19 pathology.

**Fig. 1:**
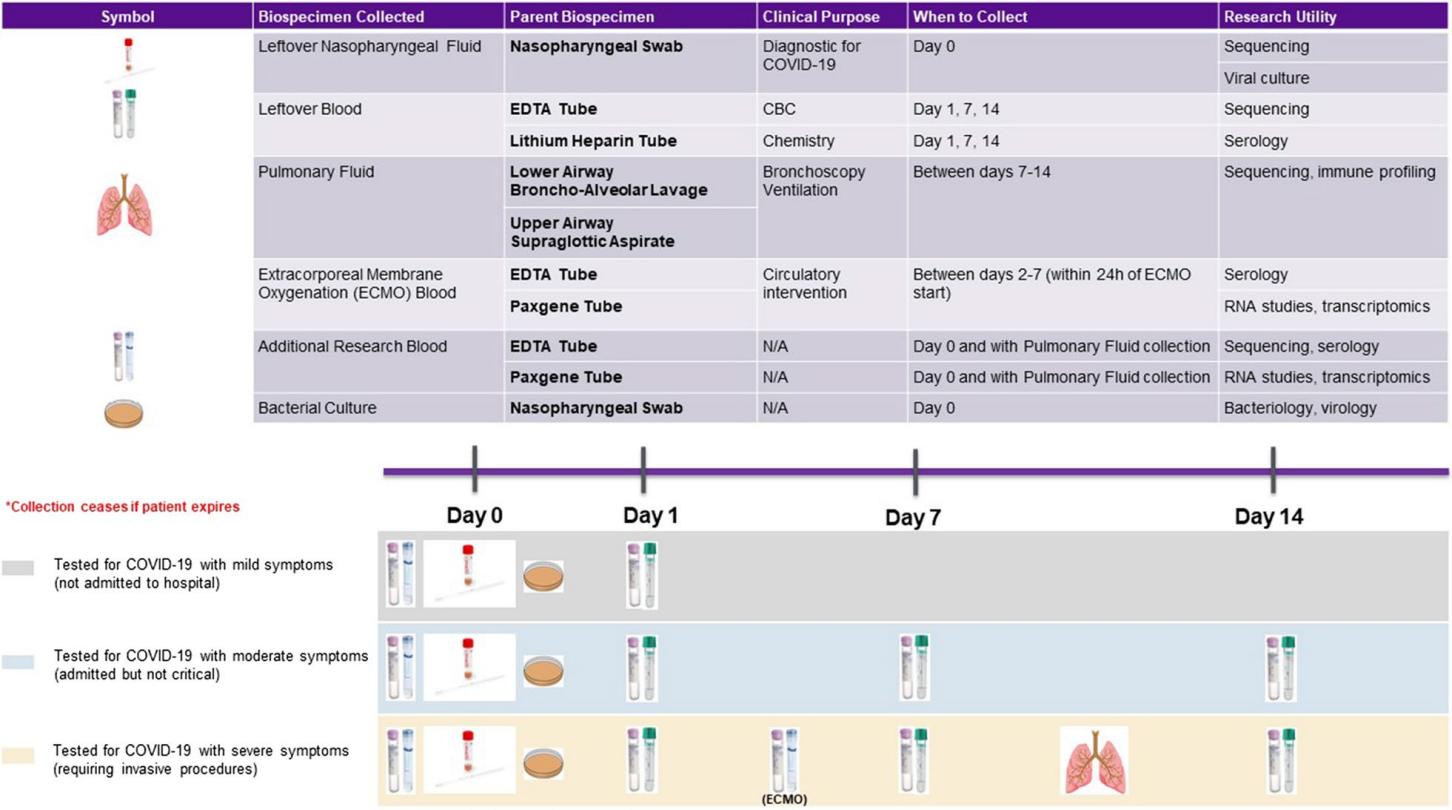
Collections and timeline for COVID-19 biospecimens at NYULH

The protocol for COVID-19 biospecimen collections for research at NYULH minimizes interruptions in the delivery of care, includes vital biosafety measures, and maximizes the research potential for banked COVID-19 biospecimens (Fig. 1). NYULH saw its first confirmed case of COVID-19 on March 11, 2020. In accordance with national and international policies, the IRB approved a waiver of consent to permit COVID-19 biospecimen collection on March 28, 2020. Within the next 3 weeks, over 10,000 patients were tested across all NYULH locations. Of the first 11,120 patients accrued to the COVID-19 database, 4267 (38.4%) tested positive and approximately 10% required hospitalization. As of April 18, 2020, we successfully collected leftover nasopharyngeal fluid from all 11,120 patients tested, and baseline and longitudinal biospecimens from 1000 hospitalized patients. By granting fast and safe collection, our timeline fosters clinical innovation that addresses the current crisis and promotes translational innovation that exploits the virus’s biology in unpredictable ways.

Centralizing governance and processing of the human samples under the CBRD also reduces exposure risk and expedites scientific progress. We therefore suggest establishing a main biorepository with institutional governance for all patients tested for COVID-19. Most importantly, this approach reduces potential exposure by diminishing transfer of biohazardous materials, limiting the number of additional specimens needed for collection, and adhering to biosafety guidelines. Further, it creates the potential to generate a holistic, statistically powered, and standardized cohort characterization by compiling all results in one bank, and it fosters important long-term follow up by overcoming the well-documented issues with decentralized biobank sustainability [6–14]. The creation of this resource at NYULH has enriched the community with high-quality patient-linked COVID-19 specimens, which has already contributed to novel findings about SARS-CoV-2 epidemiology [3].

Our amended UC protocol actively supports studies of the New York outbreak in real time and critical research to combat the pathology of COVID-19. Over the past 4 weeks, viral RNA has been extracted from 236 samples collected in this pipeline, enabling a large ongoing sequencing project that provides valuable information about the source of infections and asymptomatic spread. New sequences are continuously uploaded in the GISAID EpiCov repository (http://www.gisaid.org) as they are analyzed. This biobank pipeline also provides the necessary resources to support the clinical laboratory in the current validation of ELISA assay for antibody detection with the collection of leftover whole blood and sera from COVID-19 patients.

Biospecimen research at NYULH is continuously evolving to address the health needs of the global population that it serves. In this critical moment, we provide vital resources and recommendations for the current crisis, founded on evidence-based protocols that we amended to overcome issues faced in the heart of the global pandemic. As we continue to reveal critical findings on the recent SARS-CoV-2 outbreak, we hope to invoke a worldwide effort to build up the resources needed to eradicate COVID-19 collectively, and serve as a reminder that resilience requires adaptability even in the face of the most challenging circumstances.

## Supplementary information

**Additional file 1: Appendix S1.** Universal consent form and COVID-19 clinical database fields.

## Data Availability

Not applicable.

## Abbreviations

COVID-19: Coronavirus disease 2019
SARS-CoV-2: Severe acute respiratory syndrome coronavirus 2
NYULH: New York University Langone Health
UC: Universal consent
CBRD: Center for Biospecimen Research and Development
