# Correction to: The urgency of utilizing COVID-19 biospecimens for research in the heart of the global pandemic

**DOI:** 10.1186/s12967-021-02883-6

**Published:** 2021-06-03

**Authors:** Iman Osman, Paolo Cotzia, Una Moran, Douglas Donnelly, Carolina Arguelles-Grande, Sandra Mendoza, Andre Moreira

**Affiliations:** The New York University Langone Health (NYULH) Center of Biospecimen Research and Development, Ofce of Science and Research, NYU Grossman School of Medicine, 522 First Avenue, SML405, New York, NY 10016 USA

## Correction to: J Transl Med (2020) 18:219 10.1186/s12967-020-02388-8

In the original publication [1] there was an incomplete funding acknowledgement. In this correction article the correct and incorrect funding acknowledgement are published. The original article has been updated. The updated information is indicated in bold.

**Incorrect funding**

- This work was supported by the National Cancer Institute at the National Institutes of Health through the NYU Cancer Institute Cancer Center Support Grant (P30CA016087) and NYU Melanoma SPORE (P50CA016087) to IO.

**Correct funding**

- This work was supported by the National Cancer Institute at the National Institutes of Health through the NYU Cancer Institute Cancer Center Support Grant (P30CA016087) and **NYU Melanoma SPORE Grant (P50CA225450)** to IO.

## References

[1] Osman I, Cotzia P, Moran U, Donnelly D, Arguelles-Grande C, Mendoza S, Moreira A (2020). The urgency of utilizing COVID-19 biospecimens for research in the heart of the global pandemic. J Transl Med.

