# Supplementary material for: The urgency of utilizing COVID-19 biospecimens for research in the heart of the global pandemic
Source: J Transl Med. 2020 Jun 1;18:219. doi: 10.1186/s12967-020-02388-8 (PMC7266426; doi:10.1186/s12967-020-02388-8)
Supplement: Supplementary file 1 — Additional file 1: Appendix S1. Universal consent form and COVID-19 clinical database fields. [file 12967_2020_2388_MOESM1_ESM.docx]

**Appendix**

**Table of Contents**

Universal Consent Form……………………..…………………….....……………………………2

COVID-19 Clinical Database Fields………………………………………………………………7

**BIO-SPECIMEN UNIVERSAL CONSENT – CBRD version 2**

**Informed Consent and Authorization to Participate in Biomedical Research at the Center for Bio-specimen Research and**

**Development (CBRD)**

NYU Langone Health and its affiliated sites work together to do medical research. We look for new and improved ways to prevent and treat disease. You are invited to participate in these efforts through our NYU School of Medicine Center for Bio-specimen Research and Development (CBRD). The CBRD is a research center at NYU School of Medicine that collects tissue, blood, bodily fluids, stool samples or skin swabs. Scientists use the samples and related information to perform biomedical research.

**Taking part in this research is entirely up to you. You may consent to any part or all parts of this research or none at all.**

Your decision to participate will not affect your clinical care or health insurance coverage in any way. If you have any questions about this research or this form, you can contact the CBRD staff at (646) 501-4268 or via email at CBRDUniversalConsent@nyulangone.org.

**What research will be done?**

Researchers will study the data collected from testing and analyzing specimens provided to the CBRD that is linked to each patient’s clinical information to find ways to prevent, treat, or diagnose illness. Such studies will be designed and conducted in the future, and cannot be specifically described now. These studies may include genetic research and this may involve but is not limited to the generation of cell lines (developing a population of cells from one single cell with the same genetic makeup) and other stem cell research (under certain conditions inducing unspecialized cells to become tissue or organ specific cells with special functions).

**What will happen in this study?**

When your doctor orders tests to diagnose or treat your health condition, there may be leftover residual samples of blood, bodily fluids, tissue or stool samples. We are asking to store tissue, blood, bodily fluids (for example, urine, saliva, sputum, synovial fluid) or stool samples that are collected from you as part of your clinical care, now or in the future, that would otherwise be discarded that would be useful for future research. These samples are the leftover materials that are not needed by the laboratory performing the test that was ordered during your clinical care.

To help find new ways to diagnose, treat and prevent different conditions in the future, we would like your consent to donate a single collection of blood of up to 3 tablespoons, which is the equivalent of 45ml at one of your visits in the future that will be used for research purposes only. Alternatively, we might need to collect blood at multiple visits, as it can provide us with a better understanding of how a disease progresses. In this case, we would like your approval to collect up to 4 tablespoons or the equivalent of 60ml of blood at different times (e.g. 3, 6 or 12 months). The maximum amount of blood that can be taken for clinical research purposes cannot exceed 10 tablespoons (147ml) of blood over every 8-week period while you are obtaining treatment at

NYU Langone Health). Multiple blood sampling will only be requested by your treating physician after they have reviewed your most recent blood tests.

We would like your consent to collect additional non-invasive specimens not required for your routine clinical care that can be used for research purposes only. These samples are limited to urine, stool and skin swabs that can be collected, if needed by your physician, at any of your future visits while you are obtaining treatment at NYU Langone Health.

In order to understand how your samples relate to your other health information, we will also look at your medical records now and in the future to update your health related information, and may store some of this health information in the study database. In looking at your medical records, we may encounter sensitive health information needed for our research, including but not limited to genetic (inherited) diseases, mental health conditions (but not psychotherapy notes), alcohol or drug use problems, and HIV / AIDS. Researchers may place samples, genetic and other health information into one or more scientific databases. These may include databases maintained by the federal government. This information will not include information that directly identifies you.

**Why should I participate? What are the benefits?**

Access to more blood, bodily fluids, tissue stool samples and skin swabs permits scientists to research new and better ways to prevent, diagnose, or treat illness. While the results will not directly benefit you, your participation - at no cost to you, may help people in the future.

**What are the risks?**

There is a very small risk that your private information may be seen by someone who is not supposed to see it, even though we protect your privacy in multiple ways. There is a risk that information about taking part in genetic research may influence insurance companies and/or employers regarding your health^1^. If you agree to provide an additional blood sample, there is a very small risk of bruising or infection from drawing blood that is similar to a routine blood draw that you get from your doctor.

**Will I receive my test results including genetic results or information about my health?**

Results from any tests performed on your blood, bodily fluids and tissue samples will not be provided to you. However if we decide that the research results from your sample are clinically relevant, we will attempt to contact you to recommend appropriate followup. You and your insurer would be responsible for any follow-up tests or required care.

You should **NOT** expect to receive research results including for example genetic test results. We will not be conducting standard tests to evaluate your health. Researchers must study samples from many people over many years before they know if the results have meaning.

^1^The Genetic Information Nondiscriminatory Act of 2008 (GINA) is a federal law that protects you from discrimination based on your genetic information. It means that you cannot be deniedhealth insurance or employment based on any genetic test results. The law does not apply to life insurance or long-term care insurance.

**How will my information be kept confidential?**

There are many safeguards in place to protect your privacy. All of your identifiable health information is protected by federal law. There are certain cases where we may need to release your information to organizations such as federal and state agencies, organizations that fund or support research, or organizations that are responsible for reviewing research. Your information may be re-disclosed or used for other purposes if the person who receives your information is not required by law to protect the privacy of the information.

Your samples and related health information will be given a code number. Your name, medical record number or other information that easily identifies you will not be stored with your samples or health information. The key to the code will be stored securely in a separate file. All data will be kept in a secure manner and computer records will be password protected. If the research performed on your sample is published, it will be published without any information that directly identifies you.

Your coded samples and health information may be shared with researchers at or working with NYU School of Medicine. They may also be shared with researchers outside of NYU School of Medicine, which could include for-profit companies that are working with NYU School of Medicine researchers. Your samples will not be sold for profit. Your samples and information may be used to develop a new product or medical test to be sold. The hospital and researchers may benefit if this happens. There are no plans to pay you if your samples and information are used for this purpose.

We will only share information that identifies you with researchers if approved by an appropriate institutional review board (a group that reviews and watches over all research studies involving human subjects) or otherwise required by law.

We may share your samples and related health information with other central tissue or data banks, such as those sponsored by the U.S. National Institutes of Health, Department of Defense or for commercial purposes, so that researchers from around the country and the world can use them to study many conditions. Results or samples given to the central tissue or data banks will not contain information that directly identifies you.

**What else do I need to know?**

Your medical care or health insurance coverage will not be affected if you choose not to participate, or if you change your mind. Some researchers may develop tests, treatments or products that are worth money. You will not receive payment of any kind for your sample or for any tests, treatments, products or other things of value that may result from the research.

**What if I change my mind?**

You simply need to let us know. If you withdraw your consent, we will not collect any samples or information from you at any of your subsequent visits at NYU Langone Health. All of your samples and information that were collected up to this point that were already entered into a research study will not be removed. If you wish to withdraw your consent, please contact a member of the registration team at your next visit and let them know that you previously consented to be part of the study and would like to withdraw. The staff member will access a new version of the Bio-specimen Consent form and will select the Withdraw option. This will automatically change the responses from Yes to No to each of the questions on the form that ask for your consent. You will be asked to sign the form again indicating your desire to withdraw and a new copy of the form will be printed off for your records. Alternatively, please send an email to CBRDUniversalConsent@nyulangone.org indicating that you would like to withdraw from the study. A staff member will withdraw your name from the study so that no further samples/information can be collected by the CBRD. If you do not withdraw your consent, your samples and related health information will stay in the CBRD as long as they are useful for research. Your samples maybe kept and your medical records maybe reviewed for a long time, perhaps longer than 50 years.

**Your consent choice –** participation in this research is voluntary and if you decide not to participate, you will not incur any penalty or loss of benefits to which you are otherwise entitled. You should also note that if you do decide to participate but later discontinue participation at any time you will not incur penalty or loss of benefits to which you are otherwise entitled.

Please respond to the statements/questions below indicating whether you would like to or not like to participate

**OPTION 1:** PLEASE CONFIRM THAT YOU HAVE READ AND UNDERSTOOD THE INFORMATION ABOVE THAT DESCRIBES

THE PURPOSE OF THIS UNIVERSAL CONSENT FORM & YOU WOULD LIKE TO CONSENT TO ALL, PART OR NONE OF THIS

STUDY **□YES □ NO**

**OR**

**OPTION 2:** PLEASE CONFIRM THAT YOU HAVE PREVIOUSLY CONSENTED TO BE PART OF THIS STUDY BUT HAVE NOW

DECIDED THAT YOU WOULD LIKE TO WITHDRAW FROM FURTHER INVOLVMENT IN THIS STUDY **□WITHDRAW**

**1. CONSENT TO COLLECT, STORE & USE THE FOLLOWING BIOSPECIMENS:**

a) I give my consent to allow the collection and storage of, and research using the following types of specimens collected from me during clinical care that are not needed for other clinical purposes:

 Blood, Tissue, Bodily fluids (e.g. saliva, synovial fluid, sputum, urine), stool **□YES □ NO**

b) I give my consent to allow the collection and storage of either one single collection of blood OR multiple blood collections for research purposes only –

**PLEASE CHECK YES OR NO TO EITHER I OR II BELOW;**

i) One single collection of up to 3 tablespoons (45ml) blood. **□YES □ NO**

**OR**

ii) Multiple blood collections of up to 4 tablespoons or 60ml of blood at each visit maximum of 10 tablespoons (147ml) every 8-week period while I am obtaining treatment at NYU Langone Health. **□YES □ NO**

c) I give my consent to allow the collection and storage of stool, urine and skin swabs, if needed, while I am obtaining treatment at NYU Langone Health. **□YES □ NO**

**2. PERMISSION TO CONTACT OUTSIDE HOSPITALS/PHYSICIANS FOR PREVIOUS OR FUTURE SPECIMENS:**

I allow the researchers to contact outside hospitals, physicians or other places where I receive medical treatment in order to request specimens and related health information from those places for use in this research. **□YES □ NO**

I have read and fully understand the above information. All of my questions have been answered to my satisfaction. All blanks or deletions were filled in or completed before I signed.

If you indicated Yes to any of the questions above and sign this form, you are agreeing to take part in this research study as described to you. This means that you have read the consent form, your questions have been answered and you have decided to volunteer. You are entitled to and will be given a copy of this signed document.

**Patient Name**

**Relationship to Patient □ Self ***

**Please Select “Self” on the left.**

**Email Address**

**Witness Signature/Credentials (NYU staff member only):**

**Witness First and Last Name ***

**Witness Relationship to Patient ***

**Physician**

**Nurse**

**Other Hospital Staff**

**Comments**

**Interpreter First and Last Name and ID number**

**Interpreter Signature ***

**Date**

**Time Stored**

**Description**

**COVID-19 Clinical Database Fields:**

- Zip code (or census tract)
- Sex
- Age at dx with COVID19
- Race
- Ethnicity
- Exposure history
  - Healthcare worker
  - First responder
  - Cohabitation with other infected
  - Travel
- Past Medical History/Comorbidity
  - Hypertension
  - Diabetes
  - Coronary heart disease
  - Other heart disease/stroke
  - Chronic obstructive lung disease/asthma
  - Other chronic pulmonary disease
  - Carcinoma
  - Chronic Kidney Disease
  - Dialysis
  - HIV/AIDS
  - Cognitive impairment/dementia
  - Other
  - Pregnant (y/n)
- BMI
- Respiratory Rate
- Pulse
- Systolic blood pressure
- Smoking History (current/former/never)
- COVID diagnosis
- If COVID(+), Severity of COVID
  - Mild
  - Severe: Dyspnea (RR >30/min), hypoxia (<93% O2 sat) >50% lung involved on img in 24-48 hrs
  - Critical: respiratory failure, septic shock, multiorgan dysfunction
- Severity of symptoms
- Time of illness onset to discharge (days)
- Temperature
- Symptoms
  - Cough
  - Sputum
  - Myalgia
  - Fatigue
  - Diarrhea
  - Nausea/vomiting
  - Fever
- X-ray (Y/N)
- If yes, noted radiological findings
- Consolidation
- Ground-glass opacity
- Bilateral pulmonary infiltration
- ECHO (Y/N)
- If yes, mean pulmonary arterial pressure (Sys, Dias)
- COVID19 complications
  - Viral pneumonitis
  - Bacterial pneumonia
  - ARDS
  - Pneumothorax
  - Pleural effusion
  - COP
  - Bronchiolitis
  - Meningitis/encephalitis
  - Seizure
  - Stroke/CVA
  - CHF
  - Endo/myo/pericarditis
  - Cardiac arrhythmia
  - Cardiac ischemia
  - Cardiac arrest
- Outpatient/Hospitalization
- If hospitalized:
- Day admitted
- Length of stay
- SOFA
- qSOFA
- CURB-65
- Lab Findings
  - WBC
  - Lymphocyte count
  - Hemoglobin, g/L
  - Anemia
  - Platelet count
  - Albumin
  - ALT
  - Creatinine
  - LDH
  - Creatinine kinase
  - High sensitivity cardiac troponin
  - Prothrombin time
  - D-dimer
  - IL-6
  - Serum ferritin
  - Procalcitonin
- Treatment Received
  - Antiviral
  - Corticosteroids
  - Intravenous immunoglobulin
  - High flow nasal cannula oxygen therapy
  - Noninvasive mechanical ventilation (Y/N and duration)
  - Invasive ventilation (Y/N and duration)
  - ECMO (Y/N and duration)
  - Renal replacement therapy
  - Anti-pyretics
  - Level of Respiratory Support
  - RCT participation (Y/N, protocol number)
  - Experimental: tocilizumab, hydroxychloroquine, ritonarvir, favipiravir, remdesivir, IFN, plasmapheresis, Ribavirin, Sofosbuvir, Galidesivir, and Tenofovir, clazakizumab, therapeutic anticoagulation, dapansutrile, convalescent plasma
- Outcomes
- Survival Status/Follow up Date
